# Supplementary material for: Mutational robustness changes during long-term adaptation in laboratory budding yeast populations
Source: eLife. 2022 Jul 26;11:e76491. doi: 10.7554/eLife.76491 (PMC9355567; doi:10.7554/eLife.76491)
Supplement: Transparent reporting form [file elife-76491-transrepform1.pdf]

eLIFE
1st Floor
24 Hills Road
Cambridge CB2 1JP, UK
P 01223 855340
W elifesciences.org
T @elife

eLife's transparent reporting form

We encourage authors to provide detailed information *within their submission* to facilitate the interpretation and replication of experiments. Authors can upload supporting documentation to indicate the use of appropriate reporting guidelines for health-related research (see EQUATOR Network), life science research (see the BioSharing Information Resource), or the ARRIVE guidelines for reporting work involving animal research. Where applicable, authors should refer to any relevant reporting standards documents in this form.

If you have any questions, please consult our Journal Policies and/or contact us: editorial@elifesciences.org.

Sample-size estimation

- You should state whether an appropriate sample size was computed when the study was being designed
- You should state the statistical method of sample size computation and any required assumptions
- If no explicit power analysis was used, you should describe how you decided what sample (replicate) size (number) to use

Please outline where this information can be found within the submission (e.g., sections or figure legends), or explain why this information doesn't apply to your submission:

This is not applicable to our submission (this is an experimental study of epistasis, no power analysis or pre-experiment sample size estimation would be possible or appropriate). Sample sizes (numbers of barcodes with fitness effects measured) are all available in Supplementary File 1.

Replicates

- You should report how often each experiment was performed
- You should include a definition of biological versus technical replication
- The data obtained should be provided and sufficient information should be provided to indicate the number of independent biological and/or technical replicates
- If you encountered any outliers, you should describe how these were handled
- Criteria for exclusion/inclusion of data should be clearly stated
- High-throughput sequence data should be uploaded before submission, with a private link for reviewers provided (these are available from both GEO and ArrayExpress)

Please outline where this information can be found within the submission (e.g., sections or figure legends), or explain why this information doesn't apply to your submission:

We describe the number of clones and mutations used in this experiment in the first and second paragraph of the results, respectively. We describe the biological replication structure (clones isolated from the same population-timepoint) in these first two paragraphs as well. We discuss the barcode replication structure and outlier exclusion in the "Estimating fitness effects from barcode counts" subsection of the methods. Sequencing data and analysis code are available on NCBI and github, respectively (see Data and code accessibility).

Statistical reporting

- Statistical analysis methods should be described and justified
- Raw data should be presented in figures whenever informative to do so (typically when N per group is less than 10)
- For each experiment, you should identify the statistical tests used, exact values of N, definitions of center, methods of multiple test correction, and dispersion and precision measures (e.g., mean, median, SD, SEM, confidence intervals; and, for the major substantive results, a measure of effect size (e.g., Pearson's r, Cohen's d)
- Report exact p-values wherever possible alongside the summary statistics and 95% confidence intervals. These should be reported for all key questions and not only when the p-value is less than 0.05.

Please outline where this information can be found within the submission (e.g., sections or figure legends), or explain why this information doesn't apply to your submission:

Statistical methods are described in all subsections of the Methods text, and all analysis code is available at https://github.com/mjohnson11/VTn_pipeline . We present examples of raw fitness effect data in Figures 2-4 and show these types of plots for all mutations in Figure 3 – figure supplements 3 and 4. We report P-values, sample sizes, and relevant statistics for all model fitting in Supplementary File 1.

(For large datasets, or papers with a very large number of statistical tests, you may upload a single table file with tests, Ns, etc., with reference to sections in the manuscript.)

Group allocation

- Indicate how samples were allocated into experimental groups (in the case of clinical studies, please specify allocation to treatment method); if randomization was used, please also state if restricted randomization was applied
- Indicate if masking was used during group allocation, data collection and/or data analysis

Please outline where this information can be found within the submission (e.g., sections or figure legends), or explain why this information doesn't apply to your submission:

This is an experimental epistasis study, so we did not have experimental groups or use masking. We describe how we randomly chose populations in the "Strains" subsection of the methods.

Additional data files ("source data")

- We encourage you to upload relevant additional data files, such as numerical data that are represented as a graph in a figure, or as a summary table
- Where provided, these should be in the most useful format, and they can be uploaded as "Source data" files linked to a main figure or table
- Include model definition files including the full list of parameters used
- Include code used for data analysis (e.g., R, MatLab)
- Avoid stating that data files are "available upon request"

Please indicate the figures or tables for which source data files have been provided:

eLife Sciences Publications, Ltd is a limited liability non-profit non-stock corporation incorporated in the State of Delaware, USA, with company number 5030732, and is registered in the UK with company number FC030576 and branch number BR015634 at the address 1st Floor, 24 Hills Road, Cambridge CB2 1JP | August 2014

eLIFE
1st Floor
24 Hills Road
Cambridge CB2 1JP, UK
P 01223 855340
W elifesciences.org
T @elife

All analysis code is available at https://github.com/mjohnson11/VTn_pipeline . All sequencing data is available under bioproject PRJNA789529 on NCBI.

The data used to produce all main text figures are available in Supplementary file 1, including the parameters and coefficients of the modeling results shown in Figures 3 and 4. Supplementary file 2 contains sequences for primers used in PCRs.
